# 10-hydroxy-2-decenoic acid alleviates lipopolysaccharide-induced intestinal mucosal injury through anti-inflammatory, antioxidant, and gut microbiota modulation activities in chickens

**DOI:** 10.3389/fmicb.2023.1285299

**Published:** 2023-10-17

**Authors:** Lianquan Han, Maolu Zhang, Fuwei Li, Jing Su, Ruiming Wang, Guiming Li, Xiaohui Yang

**Affiliations:** ^1^State Key Laboratory of Biobased Material and Green Papermaking (LBMP), Qilu University of Technology (Shandong Academy of Sciences), Jinan, China; ^2^Institute of Poultry Science, Shandong Academy of Agricultural Sciences, Jinan, China

**Keywords:** 10-hydroxy-2-decenoic acid, lipopolysaccharide, intestinal barrier, inflammation, antioxidant, gut microbiota

## Abstract

**Introduction:**

This study aimed to investigated the effects of 10-hydroxy-2-decenoic acid (10-HDA) on the growth performance, intestinal barrier, inflammatory response, oxidative stress, and gut microbiota of chickens challenged with lipopolysaccharide (LPS).

**Methods:**

A total of 240 one-day-old chickens were randomly assigned to five treatment groups: (1) control group (basal diet + saline); (2) LPS group (basal diet + LPS); (3) Chlortetracycline (CTC) group (basal diet containing 75 mg/kg CTC + LPS); (4) 0.1% 10-HDA group (basal diet containing 1 g/kg 10-HDA + LPS); and (5) 0.5% 10-HDA group (basal diet containing 5 g/kg 10-HDA + LPS). All chickens were injected intraperitoneally with 0.5 mg/kg body weight of either LPS or saline at 17, 19, and 21 days of age.

**Results:**

The results showed that dietary 10-HDA supplementation attenuated the loss in growth performance caused by the LPS challenge (*p* < 0.05). 10-HDA effectively alleviated LPS-induced intestinal mucosal injury, as evidenced by reduced bleeding, decreased serum diamine oxidase levels (*p* < 0.05), and increased villus/crypt ratios of the jejunum and ileum (*p* < 0.05). Dietary treatment with 0.1% 10-HDA reduced the concentrations of inflammatory cytokines (TNF-α, IL-1β, IL-6; *p* < 0.05), and increased immunoglobulin (IgA, IgG) and antioxidant enzyme levels (CAT, GSH-px, T-SOD) in the serum of LPS-challenged chickens (*p* < 0.05). These effects were similar to those observed in the CTC group. Moreover, 0.1% 10-HDA treatment reversed the LPS-induced variations in the mRNA expression of genes related to inflammation, antioxidant capacity, and intestinal tight junctions (*p* < 0.05). 16S rRNA analysis revealed that 10-HDA supplementation increased the relative abundance of *Faecalibacterium* and *Clostridia_UCG-014* (*p* < 0.05). Additionally, it decreased the abundance of *Clostridia_vadinBB60_group*, *Eubacterium_nodatum_group*, and *UC5-1-2E3* (*p* < 0.05). These changes were correlated with reduced inflammation and improved antioxidant capacity in the LPS-challenged chickens.

**Conclusion:**

Collectively, dietary 10-HDA supplementation alleviated LPS-induced intestinal mucosal injury and the loss of growth performance through anti-inflammatory, antioxidant, and gut microbiota modulation activities in chickens. Moreover, 0.1% 10-HDA supplementation had comparable or even better protection for LPS-challenged chickens than supplementation with antibiotics or 0.5% 10-HDA. 10-HDA has the potential to be used as an alternative to antibiotics in protecting the intestinal health and improving the performance of poultry.

## 1. Introduction

The intestinal tract is not only the main organ for the digestion and absorption of nutrients in animals, but also acts as an innate barrier to maintain body homeostasis (Vancamelbeke and Vermeire, 2017). Modern intensive production makes poultry more susceptible to various stress factors, such as environmental, nutritional, bacterial, and toxin infections, which may destroy the intestinal barrier function and cause intestinal and systemic diseases in poultry (Yang et al., 2011; Adedokun and Olojede, 2019). Lipopolysaccharide (LPS) is an outer membrane component of Gram-negative bacteria. LPS-mediated intestinal barrier dysfunction increases intestinal permeability, causing systemic inflammatory responses and imbalances in the oxidative and antioxidant defense systems (Chen Y. P. et al., 2018; Zhang et al., 2020; Tao et al., 2022). Therefore, intraperitoneal injection of LPS in animals serves as an effective model to study intestinal mucosal damage and inflammation (Leshchinsky and Klasing, 2001). Antibiotics have been widely used in animal production as feed additives to reduce stress and promote growth (Mehdi et al., 2018). Nevertheless, prolonged antibiotic usage leads to the presence of antibiotic residues, drug resistance, and environmental pollution (Bacanli and Basaran, 2019; Robles-Jimenez et al., 2022). Hence, it is urgent to explore new feed additives that can replace antibiotics in the poultry industry.

10-Hydroxy-2-decenoic acid (10-HDA), an unsaturated medium-chain fatty acid with terminal hydroxylation, is the main fatty acid component in royal jelly. It has various physiological activities, such as antibacterial (Melliou and Chinou, 2005; Gao et al., 2022), anti-inflammatory (Chen Y. F. et al., 2018; You et al., 2020), antioxidant (Gui et al., 2018), and immunomodulation activities (Fan et al., 2020). *In-vitro* studies conducted on murine macrophages and human colon cancer cells have demonstrated that 10-HDA exerts anti-inflammatory activities through inhibition of the NF-κB pathway and reduction of proinflammatory cytokine secretion (Sugiyama et al., 2012; Yang et al., 2018). Furthermore, 10-HDA mitigates DSS-induced colitis in mice through regulation of the NLRP3 inflammasome-mediated pyroptotic pathway and enhancement of the colonic barrier function (Huang et al., 2022). 10-HDA can also resist oxidative stress by increasing antioxidant oxidase activity and reducing ROS production (Hu X. et al., 2022). Previously, the low yield and high cost of the physical extraction and chemical synthesis of 10-HDA have limited its application. Recently, Li et al. reported the production of 10-HDA by microbial whole-cell catalysis utilizing synthetic biology strategies (Li et al., 2022), which has laid the foundation for the application of 10-HDA in the poultry industry.

A previous study showed that 10-HDA improves the growth performance, immune function, and antioxidant capacity of broilers under non-stress conditions (Zhang et al., 2022). However, the effects of 10-HDA on the intestinal health and the gut microbiota composition in chickens under stressful conditions remain unclear. Therefore, this study aimed to determine the effects of dietary 10-HDA supplementation on the intestinal barrier, inflammatory responses, and oxidative stress, as well as to investigate the potential correlations with alterations in the gut microbiota in chickens subjected to LPS challenge. This study provides valuable references for the application of 10-HDA in protecting intestinal health and improving the performance of poultry.

## 2. Materials and methods

### 2.1. Experimental design

A total of 240 one-day-old male chickens (Hy-Line Brown) with similar body weights were randomly assigned to five groups, with six replicates per group and eight chickens per replicate. The five groups were as follows: (1) CON group, basal diet + saline challenge; (2) LPS group, basal diet + LPS challenge; (3) CTC group, basal diet containing 75 mg/kg chlortetracycline (CTC) + LPS challenge; (4) 0.1% 10-HDA group, basal diet containing 1 g/kg 10-HDA + LPS challenge; and (5) 0.5% 10-HDA group, basal diet containing 5 g/kg 10-HDA + LPS challenge. 10-HDA (purity ≥98%) was purchased from Shanghai Deepak Biotechnology Co., Ltd. (Shanghai, China). The *Escherichia coli* LPS was dissolved in 0.86% sterile saline. On day 17, 19, and 21, the chickens were intraperitoneally injected with LPS (0.5 mg/kg body weight) or an equivalent amount of saline. All chickens were housed in an environmentally controlled room. The temperature was maintained at 33 to 35°C in the first week, and then gradually decreased to 26°C at 21 days of age. Continuous light was provided throughout the experimental period. All chickens had *ad-libitum* access to feed and fresh water. The composition and nutrient levels of the basal diet are shown in Supplementary Table S1. The basal diet fulfilled the nutritional requirements of chickens (NY/T33-2004, China).

### 2.2. Growth performance measurement

The body weight and feed intake of all chickens were recorded on day 1 and 21 to determine the average daily gain (ADG) and the average daily feed intake (ADFI). The feed-to-gain ratio (F/G) was defined as ADFI:ADG.

### 2.3. Sample collection

Three hours after the LPS injection on day 21, one chicken whose body weight was close to the average body weight for each replicate (six chickens per treatment group) was selected for sampling. Blood was collected from the wing vein, and the serum was obtained via centrifugation at 3,000 × *g* for 15 min at 4°C. The chickens were euthanized via artificial cervical dislocation. Duodenum, jejunum, and ileum specimens were taken from the mesenteric tissue and photographed. An intestinal segment with a size of 2 cm was dissected from both the jejunum and ileum and fixed in 4% paraformaldehyde for histological analysis. Jejunal mucosa was scraped and stored at −80°C for gene expression analysis. Cecal contents were collected for subsequent 16S rRNA sequencing.

### 2.4. Intestinal morphometry

After fixation, the jejunal and ileal segments were dehydrated using an ethanol gradient, cleared using dimethylbenzene, and subsequently embedded in paraffin. The tissues were sliced and subjected to hematoxylin and eosin (HE) staining. Images were acquired using an Eclipse 80i microscope (Nikon Inc., Tokyo, Japan). The villus height (VH) and crypt depth (CD) were determined using the Image-Pro Plus 6.0 software, and the VH/CD ratios (VCR) were calculated.

### 2.5. Analysis of the serum biochemical status

The serum levels of diamine oxidase (DAO), tumor necrosis factor-α (TNF-α), interleukin (IL)-1β, IL-6, immunoglobulin (Ig) A, IgG, catalase (CAT), total superoxide dismutase (T-SOD), and glutathione peroxidase (GSH-px) were determined by chicken ELISA kits (Hengyuan Biotech. Co., Shanghai, China) according to the manufacturer’s protocols.

### 2.6. Real-time quantitative PCR (RT-qPCR)

Total RNA was extracted from the jejunal mucosa using TRI pure reagent (Aidlab Biotech. Co., Beijing, China). For each sample, cDNA was synthesized with 1 μg of total RNA using a SPARKscript II RT Plus Kit (SparkJade Biotech. Co., Jinan, China) following the manufacturer’s protocol. RT-qPCR was conducted using a real-time PCR system and SYBR Green qPCR Mix (Vazyme, Nanjing, China). The relative mRNA expression levels were analyzed using the 2^-ΔΔCT^ method. The gene-specific primers are shown in Supplementary Table S2, and β-actin was used as the internal standard.

### 2.7. Analysis of the cecal microbiota composition

The microbial DNA of the cecal contents was extracted using the EZNA^®^ soil DNA Kit (Omega Bio-tek, Norcross, GA, United States). The V3–V4 variable region of the bacterial 16S rRNA gene was amplified using primers 338F (5′-ACTCCTACGGGAGGCAGCAG-3′) and 806R (5′-GGACTACHVGGGTWTCTAAT-3′) (Liu et al., 2016). After purification, amplicons were collected and subjected to paired-end sequencing using an Illumina MiSeq PE300 platform (Illumina, CA, United States). The raw FASTQ files underwent quality filtering and merging using FLASH 1.2.11. Subsequently, operational taxonomic units (OTUs) were derived by clustering the optimized sequences at a 97% identity threshold. The taxonomy of the OTU sequences was determined using the RDP Classifier (version 2.13) and the 16S rRNA gene database. Bioinformatic analysis of the cecal microbiota was performed on the Majorbio Cloud platform.^1^

### 2.8. Statistical analysis

Data were analyzed using SPSS 26.0 (SPSS Inc., Armonk, NY, USA). Variations among groups were compared using one-way ANOVA, followed by Duncan’s multiple range test. Data are presented as the mean ± standard error of the mean (SEM). Differences were considered significant at *p* < 0.05.

## 3. Results

### 3.1. Growth performance

The effects of 10-HDA supplementation on the growth performance of LPS-challenged chickens are shown in Table 1. The LPS challenge resulted in a significant decrease in ADFI (*p* < 0.05) and ADG (*p* < 0.05), as well as in an increase in the feed to gain ratio of chickens from 1 to 21 days (*p* < 0.05) when compared to the control group. Dietary addition of 0.1 and 0.5% 10-HDA significantly reduced the feed-to-gain ratio of the LPS-challenged chickens (*p* < 0.05), but had no significant effects on ADFI or ADG during the experimental period (*p* > 0.05). The CTC supplementation did not show any significant effect on the LPS-induced decline in the growth performance of chickens (*p* > 0.05).

**Table 1:** Effects of dietary 10-HDA supplementation on the growth performance of LPS-challenged chickens.

Items^1^	Treatments^2^					SEM	*p*-value
	CON	LPS	CTC	0.1% 10-HDA	0.5% 10-HDA		
ADFI (g/d)	15.76^a^	15.27^b^	15.11^b^	15.12^b^	15.15^b^	0.075	0.017
ADG (g/d)	8.87^a^	8.24^b^	8.27^b^	8.41^b^	8.39^b^	0.061	0.001
F/G	1.78^a^	1.85^c^	1.83^bc^	1.80^ab^	1.81^ab^	0.008	0.014

^a, b, c^ Means with different superscripts within a row differ significantly (*p* < 0.05, *n* = 6). ^1^ADFI, average daily feed intake; ADG, average daily gain; F/G, feed-to-gain ratio. ^2^CON, basal diet + saline challenge; LPS, basal diet + LPS challenge; CTC, basal diet containing 75 mg/kg chlortetracycline + LPS challenge; 0.1% 10-HDA, basal diet containing 1 g/kg 10-HDA + LPS challenge; 0.5% 10-HDA, basal diet containing 5 g/kg 10-HDA + LPS challenge.

### 3.2. Intestinal morphology and permeability

**Figure 1:**
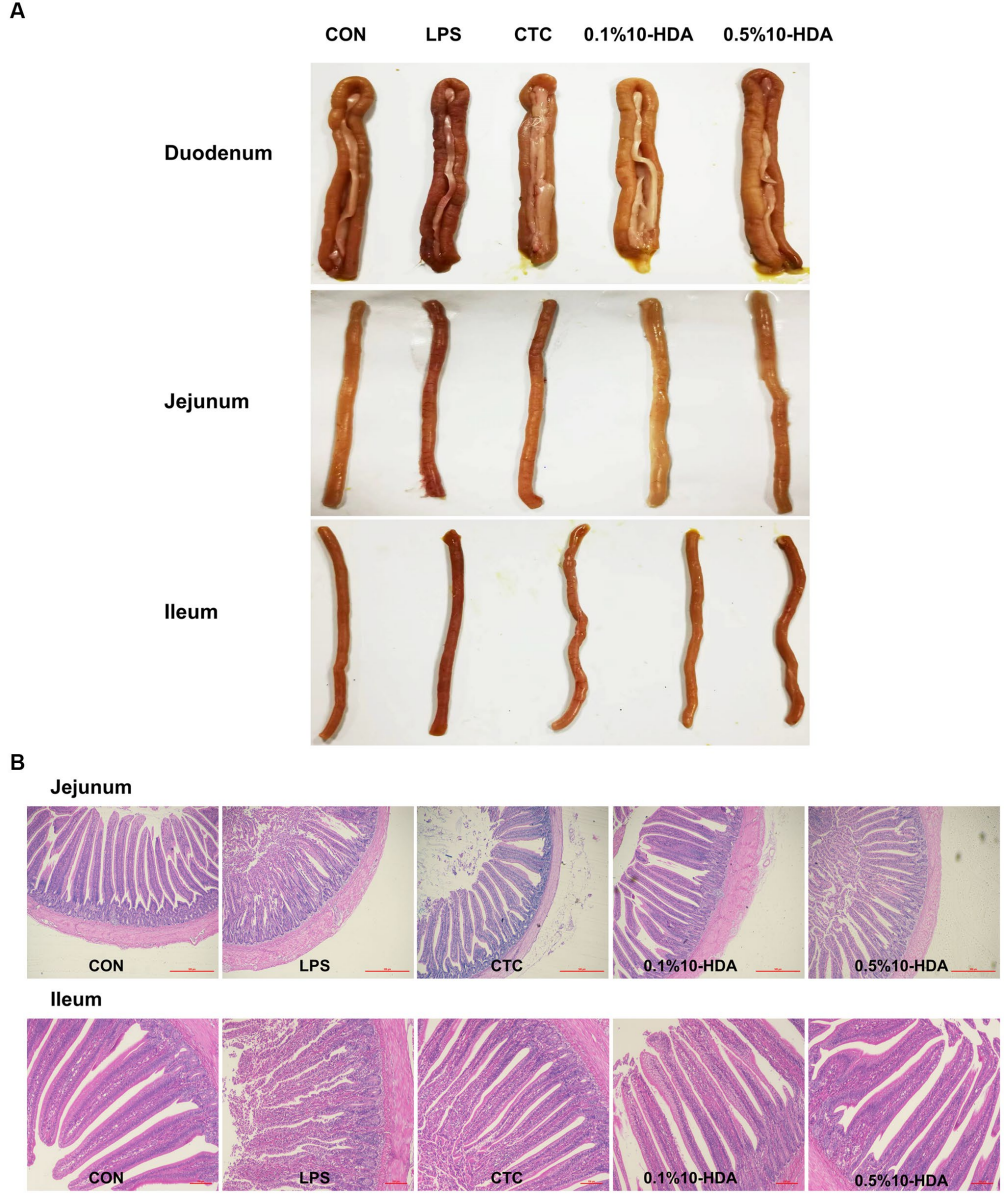
Effect of 10-HDA supplementation on the intestinal morphology of LPS-challenged chickens. **(A)** Photographs of the duodenum, jejunum, and ileum. **(B)** Morphological structure of the jejunum and ileum by HE staining. Scale bar 500 μm, 40×. Scale bar 100 μm, 100 ×. CON, basal diet + saline challenge; LPS, basal diet + LPS challenge; CTC, basal diet containing 75 mg/kg chlortetracycline + LPS challenge; 0.1% 10-HDA, basal diet containing 1 g/kg 10-HDA + LPS challenge; 0.5% 10-HDA, basal diet containing 5 g/kg 10-HDA + LPS challenge.

The intestinal morphology of chickens is shown in Figure 1A. After the LPS challenge, the duodenum, jejunum, and ileum showed obvious bleeding spots. Dietary addition of CTC or 10-HDA effectively alleviated the bleeding, and the 0.1% 10-HDA treatment appeared to be more effective than the CTC and 0.5% 10-HDA treatments. The HE staining results revealed that the epithelial villi of the jejunum and ileum were severely damaged, and a large area of the intestinal villi epithelium was shed in response to the LPS challenge, whereas the CTC or 10-HDA treatments alleviated this damage (Figure 1B). Moreover, the integrity of the intestinal barrier was determined by measuring the activity of DAO in the serum. The LPS challenge resulted in a notable increase in the serum DAO levels compared to those of the control (*p* < 0.05). In contrast, the addition of CTC or 10-HDA markedly reduced the LPS-induced elevation in the serum DAO levels (*p* < 0.05). Moreover, no significant differences were observed between the 10-HDA and CTC groups (*p* > 0.05; Table 2).

**Table 2:** Effects of dietary 10-HDA supplementation on serum DAO levels and the intestinal histomorphology of LPS-challenged chickens.

Items^1^	Treatments^2^						
	CON	LPS	CTC	0.1% 10-HDA	0.5% 10-HDA	SEM	*p*-value
Serum							
DAO (U/L)	12.36^b^	15.99^a^	8.59^b^	11.85^b^	14.18^b^	1.699	0.015
Jejunum							
VH (μm)	711.47	765.47	838.80	825.97	856.35	82.58	0.427
CD (μm)	95.93^b^	184.73^a^	115.96^b^	97.34^b^	116.33^b^	15.04	<0.001
VCR	7.41^a^	4.24^b^	7.23^a^	8.50^a^	7.35^a^	0.44	<0.001
Ileum							
VH (μm)	600.36^ab^	492.91^b^	614.05^ab^	631.09^a^	605.31^ab^	37.74	<0.001
CD (μm)	98.75	122.51	101.36	104.35	98.48	13.66	0.418
VCR	6.09^a^	4.05^b^	6.11^a^	6.12^a^	6.31^a^	0.548	0.010

^a, b^Means with different superscripts within a row differ significantly (*p* < 0.05, *n* = 6). ^1^DAO, diamine oxidase; VH, villus height; CD, crypt depth; VCR, villus height/ crypt depth ratio. ^2^CON, basal diet + saline challenge; LPS, basal diet + LPS challenge; CTC, basal diet containing 75 mg/kg chlortetracycline + LPS challenge; 0.1% 10-HDA, basal diet containing 1 g/kg 10-HDA + LPS challenge; 0.5% 10-HDA, basal diet containing 5 g/kg 10-HDA + LPS challenge.

As shown in Table 2, LPS challenge significantly increased the CD in the jejunum (*p* < 0.05) and decreased the VCR of the jejunum and ileum (*p* < 0.05). In contrast, dietary 10-HDA supplementation significantly decreased the CD in the jejunum (*p* < 0.05) and increased the VCR of the jejunum and ileum compared to the LPS group (*p* < 0.05). These effects were similar to those observed in the CTC group (*p* > 0.05). Additionally, 0.1% 10-HDA supplementation significantly increased the ileal VH (*p* < 0.05). The results indicate that 10-HDA could replace antibiotics to alleviate LPS-induced injury to the intestinal mucosal structure.

### 3.3. Serum cytokines, immunoglobulin levels, and oxidation status

**Figure 2:**
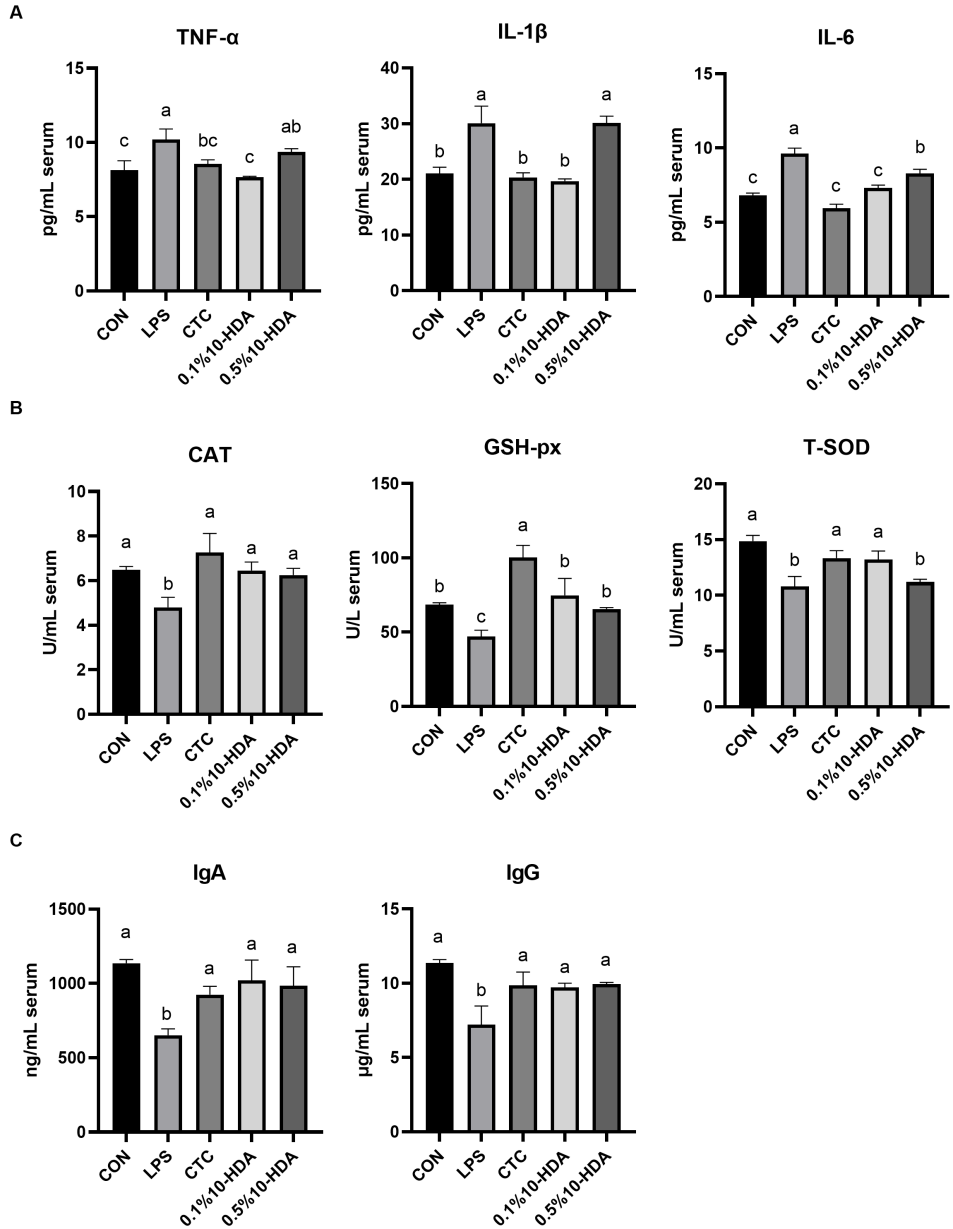
Effect of 10-HDA on the serum biochemical indices of LPS-challenged chickens. **(A)** Serum levels of inflammatory cytokines. **(B)** Activity of antioxidant enzymes. **(C)** Serum concentrations of immunoglobulins. Grouping information is the same as that in Figure 1. ^a, b, c^ Means with different superscripts are significantly different (*p* < 0.05).

The effects of 10-HDA supplementation on the inflammatory cytokine and immunoglobulin levels and antioxidant enzyme activities in the serum of LPS-challenged chickens are shown in Figure 2. LPS challenge significantly increased the serum levels of cytokines TNF-α, IL-1β, and IL-6 (*p* < 0.05), whereas CTC or 0.1% 10-HDA supplementation significantly dampened the increase in the levels of these cytokines (*p* < 0.05). Moreover, no significant differences were observed between the 0.1% 10-HDA group and the CTC group (*p* > 0.05). Compared to the LPS group, the 0.5% 10-HDA treatment reduced IL-6 levels (*p* < 0.05), but had no significant effect on TNF-α and IL-1β levels (*p* > 0.05) (Figure 2A). Figure 2B shows that LPS challenge decreased the activities of CAT, GSH-px, and T-SOD in the serum (*p* < 0.05), whereas the CTC or 0.1% 10-HDA pretreatment significantly increased their activities (*p* < 0.05). Furthermore, treatment with 0.5% 10-HDA increased CAT and GSH-px activities (*p* < 0.05), but had no significant effect on T-SOD activity (*p* > 0.05). As shown in Figure 2C, LPS challenge reduced serum IgA and IgG concentrations (*p* < 0.05), whereas the CTC or 10-HDA treatments significantly reversed this decrease (*p* < 0.05). These results indicate that dietary supplementation with 10-HDA, particularly at a concentration of 0.1%, could effectively alleviate LPS-induced inflammation and oxidative stress in chickens.

### 3.4. Inflammation-related gene expression

**Figure 3:**
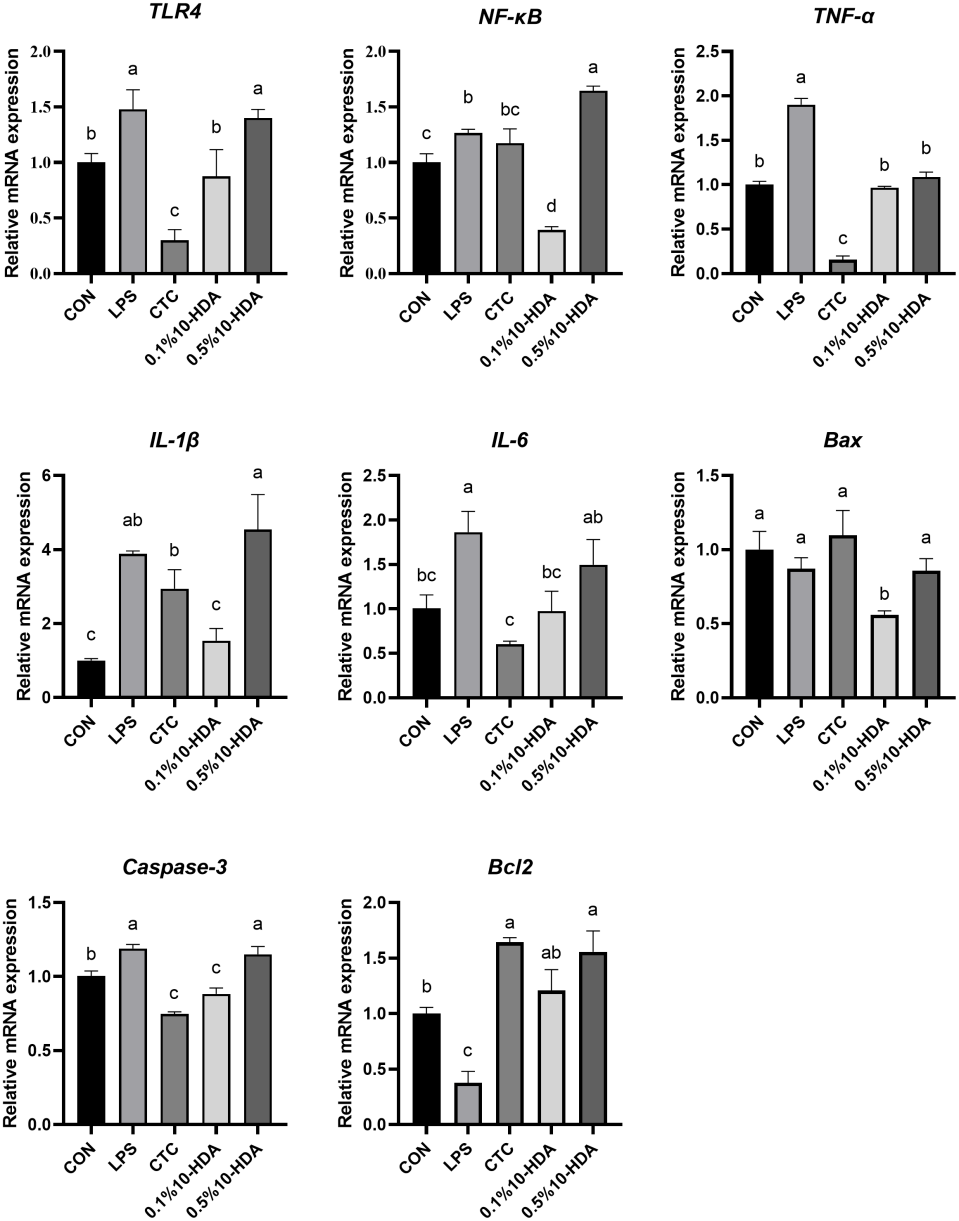
Effect of 10-HDA on the mRNA expression of inflammatory cytokines and apoptosis genes in the jejunum of chickens challenged with LPS. Grouping information is the same as that in Figure 1. ^a, b, c, d^ Means with different superscripts are significantly different (*p* < 0.05).

Quantitative PCR was used to investigate the mRNA expression of inflammatory cytokines and apoptotic regulators in the chicken jejunum (Figure 3). The results revealed that LPS challenge dramatically enhanced the expression levels of *TLR4*, *NF-κB*, *IL-1β*, *IL-6*, *TNF-α*, and *Caspase-3* (*p* < 0.05), whereas 0.1% 10-HDA supplementation mitigated the up-regulation of these genes (*p* < 0.05). CTC supplementation decreased the expression of *TLR4*, *IL-6*, and *TNF-α* compared to the LPS group (*p* < 0.05), but had no considerable effect on *NF-κB* and *IL-1β* expression (*p* > 0.05). Treatment with 0.5% 10-HDA decreased the expression of *TNF-α* (*p* < 0.05), increased the expression of *NF-κB* (*p* < 0.05), and had no significant effect on *TLR4, IL-1β, IL-6, Bax,* and *Caspase-3* levels (*p* > 0.05). LPS challenge decreased the expression of the antiapoptotic gene *Bcl2* (*p* < 0.05), whereas treatment with CTC or 10-HDA significantly increased *Bcl2* expression (*p* < 0.05). These results further demonstrate the superiority of 0.1% 10-HDA supplementation in alleviating LPS-induced intestinal inflammation in chickens.

### 3.5. Expression of antioxidant and tight junction genes

**Figure 4:**
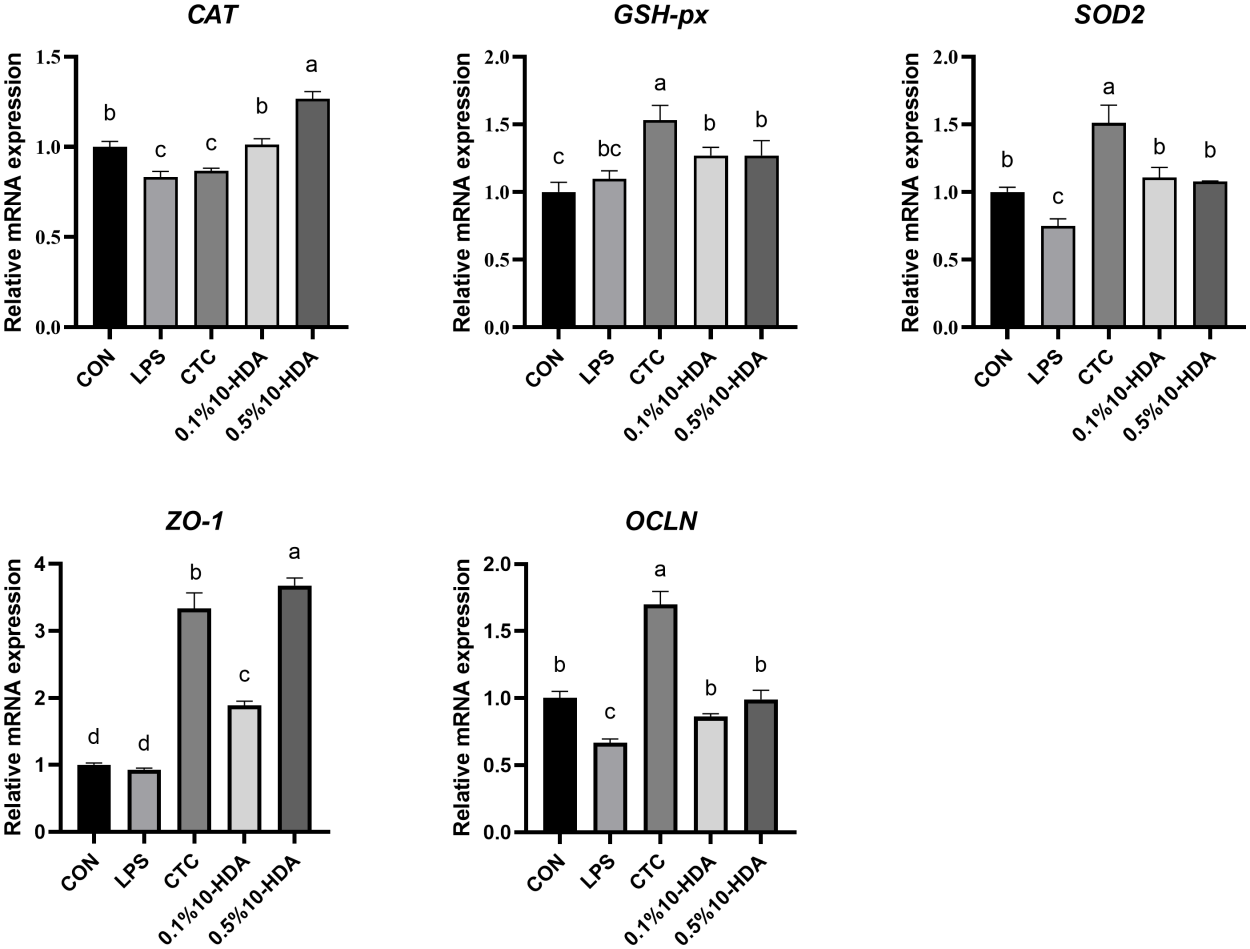
Effect of 10-HDA on the mRNA expression of antioxidant and tight junction genes in the jejunum of LPS-challenged chickens. Grouping information is the same as that in Figure 1. ^a, b, c, d^ Means with different superscripts are significantly different (*p* < 0.05).

The mRNA expression of antioxidant and tight junction genes in the jejunum of chickens is presented in Figure 4. LPS stimulation reduced the expression of *CAT*, *SOD2*, and *OCLN* (*p* < 0.05). Compared with those of the LPS group, the expression levels of *CAT*, *SOD2*, *ZO-1,* and *OCLN* were significantly increased by the supplementation with 0.1 and 0.5% 10-HDA (*p* < 0.05). The CTC treatment enhanced the expression levels of *GSH-px*, *SOD2*, *ZO-1,* and *OCLN* (*p* < 0.05).

### 3.6. Cecal microbial composition

**Figure 5:**
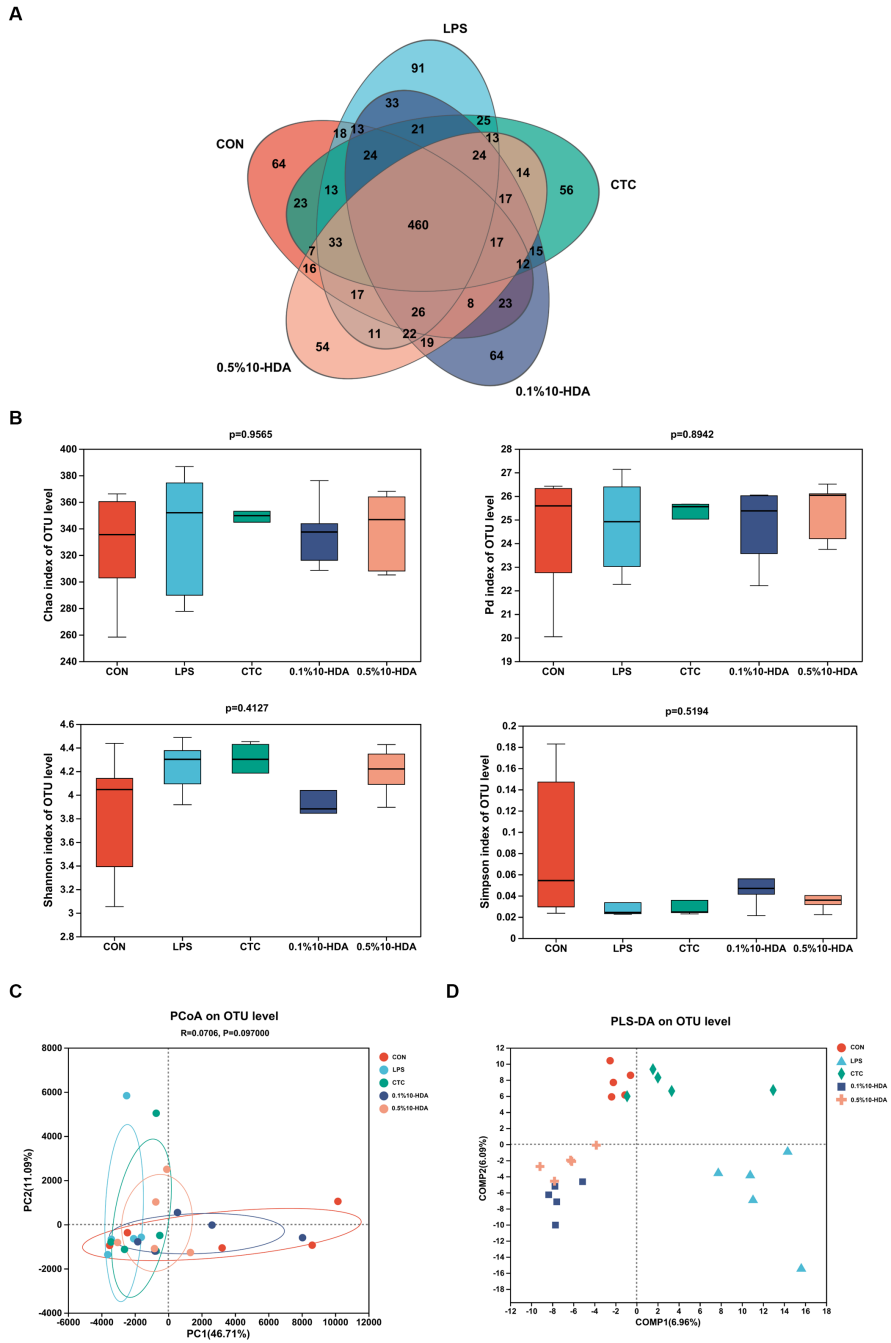
Effects of dietary 10-HDA supplementation on the cecal microbiota diversity of LPS-challenged chickens. **(A)** Venn diagram. **(B)** Alpha diversity index analysis. **(C)** PCoA based on the Euclidean distance. **(D)** Partial least squares discriminant analysis (PLS-DA). Grouping information is the same as that in Figure 1.

We investigated the effects of 10-HDA on the cecal microbiota of chickens treated with LPS using 16S rRNA high-throughput sequencing. The Venn diagram in Figure 5A shows that the total OTUs in the CON, LPS, CTC, 0.1% 10-HDA, and 0.5% 10-HDA groups were 774, 844, 774, 798, and 758, respectively, and 460 mutual OTUs were detected among the five groups. The Chao1, Faith PD, Shannon, and Simpson indices were calculated to assess the alpha diversity of the samples. However, no significant variations were detected among the groups (Figure 5B). PCoA and PLS-DA analysis showed that the samples of the LPS group were clearly separated from those of the CON group, suggesting that the cecal microbial community of the LPS group was distinct from that of the CON group (Figures 5C,D). The distribution of samples in the 0.1% 10-HDA group was similar to that of the CON group.

**Figure 6:**
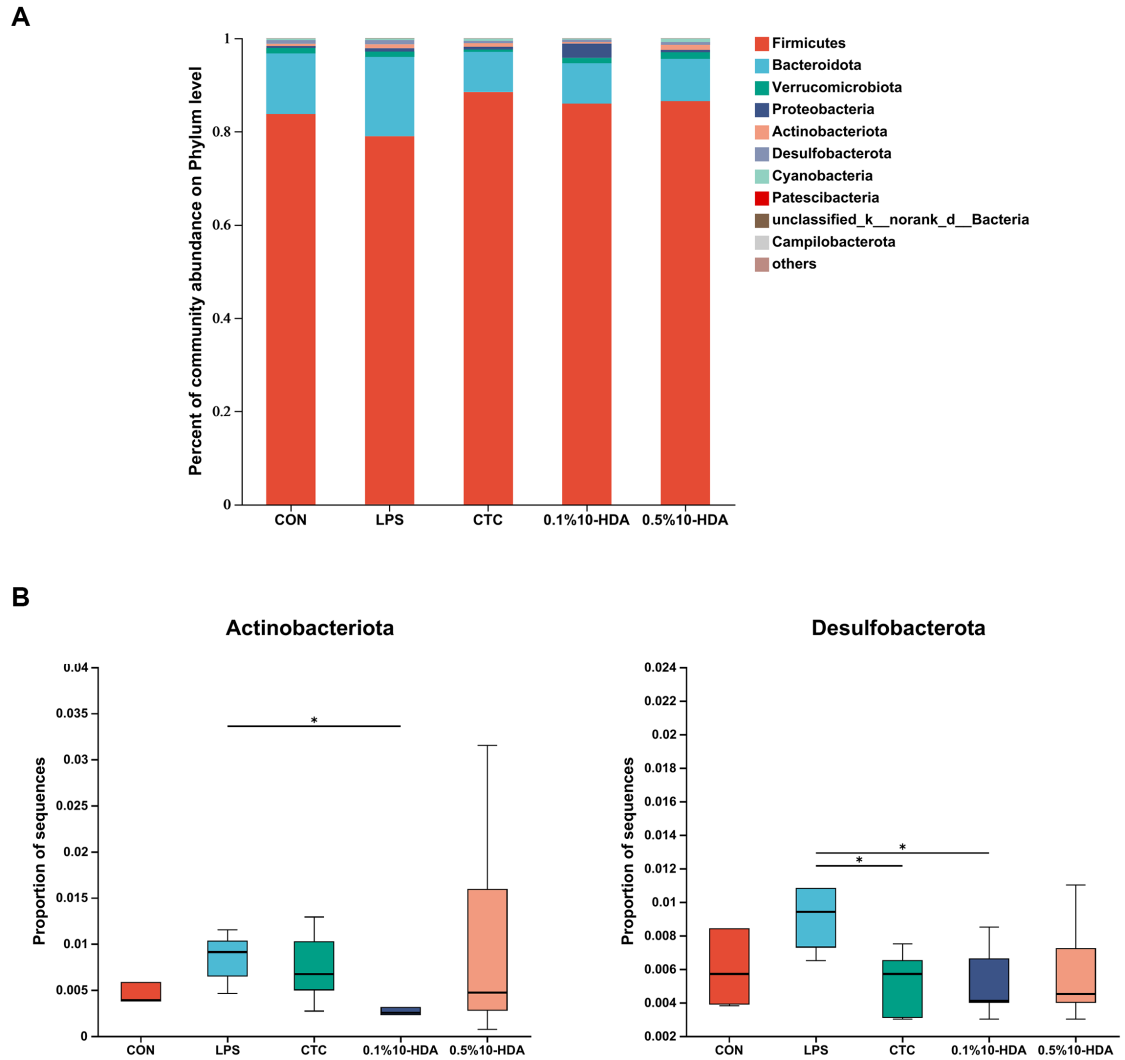
Effects of 10-HDA on the cecal microbiota composition in LPS-challenged chickens at the phylum level. **(A)** Gut microbiota composition. **(B)** Analysis of differences in abundance among groups. Grouping information is the same as that in Figure 1; *p < 0.05, **p < 0.01.

**Figure 7:**
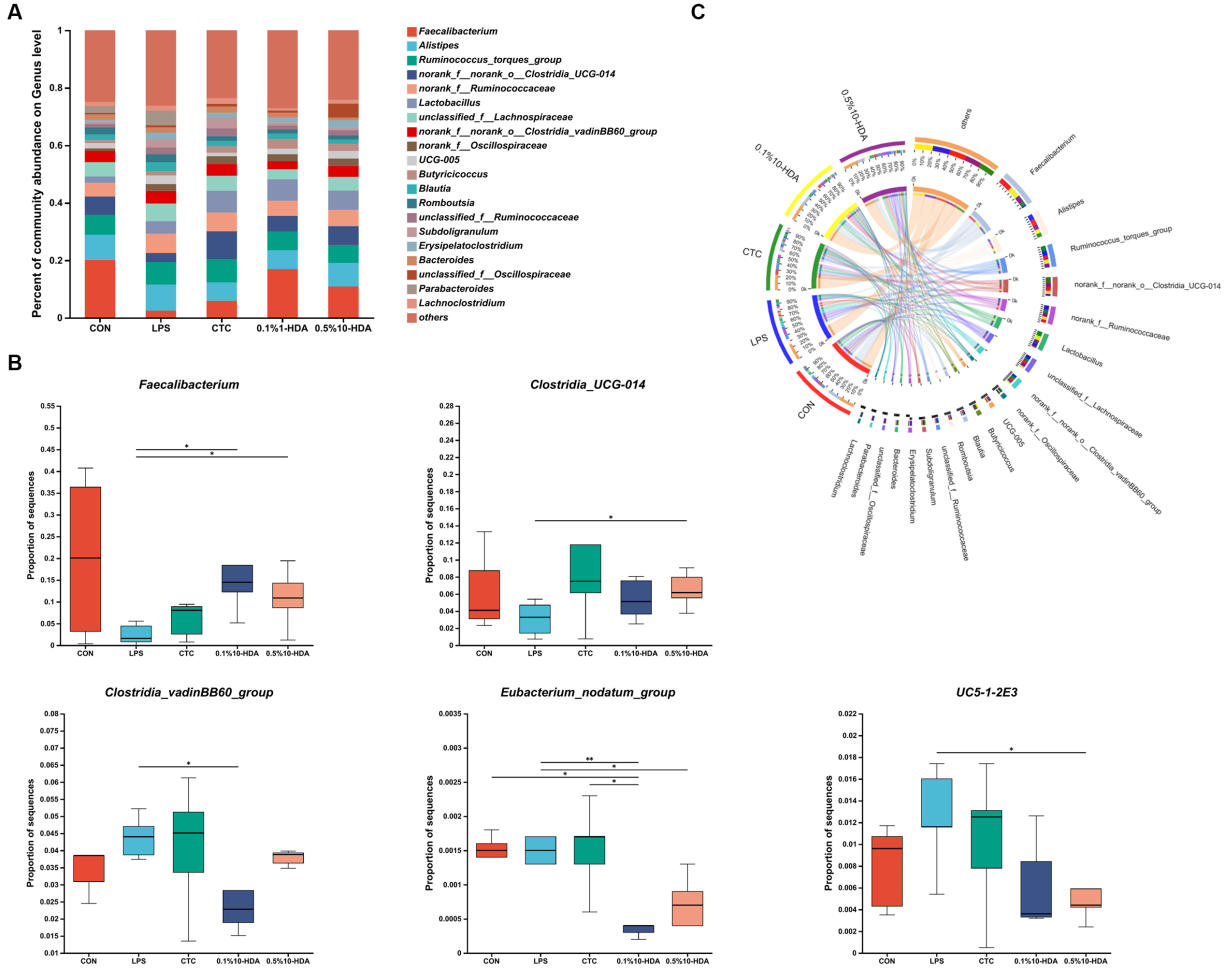
Effects of 10-HDA on the cecal microbiota composition of LPS-challenged chickens at the genus level. **(A)** Gut microbiota composition. **(B)** Analysis of differences in abundance among groups. **(C)** Circos diagram showing the dominant genera in each group. Grouping information is the same as that in Figure 1; *p < 0.05, **p < 0.01.

Figure 6 shows the differences in microbial composition at the phylum level. The results indicate that Firmicutes and Bacteroidetes were the predominant intestinal phyla in all groups. Dietary 0.1% 10-HDA supplementation decreased the proportion of Actinobacteria and Desulfobacterota in the LPS-challenged chickens (*p* < 0.05). At the genus level, we analyzed the abundance of the community of the 20 dominant genera within each group (Figure 7A). Compared with the LPS group, the 0.1% 10-HDA treatment significantly increased the abundance of *Faecalibacterium* (*p* < 0.05) and reduced the abundance of *Clostridia_vadinBB60_group* (*p* < 0.05) and *Eubacterium_nodatum* (*p* < 0.01). Dietary 0.5% 10-HDA increased the abundance of *Faecalibacterium* (*p* < 0.05) and *Clostridia_UCG-014* (*p* < 0.05), and reduced the abundance of *Eubacterium_nodatum* (*p* < 0.05) and *UC5-1-2E3* (*p* < 0.05) (Figure 7B). Furthermore, the Circos diagram illustrates that the proportion of *Lactobacillus* and *Butyricicoccus* in the 0.1 and 0.5% 10-HDA groups was higher than that in the LPS group. *Alistipes* was the dominant genus in the LPS group (Figure 7C).

**Figure 8:**
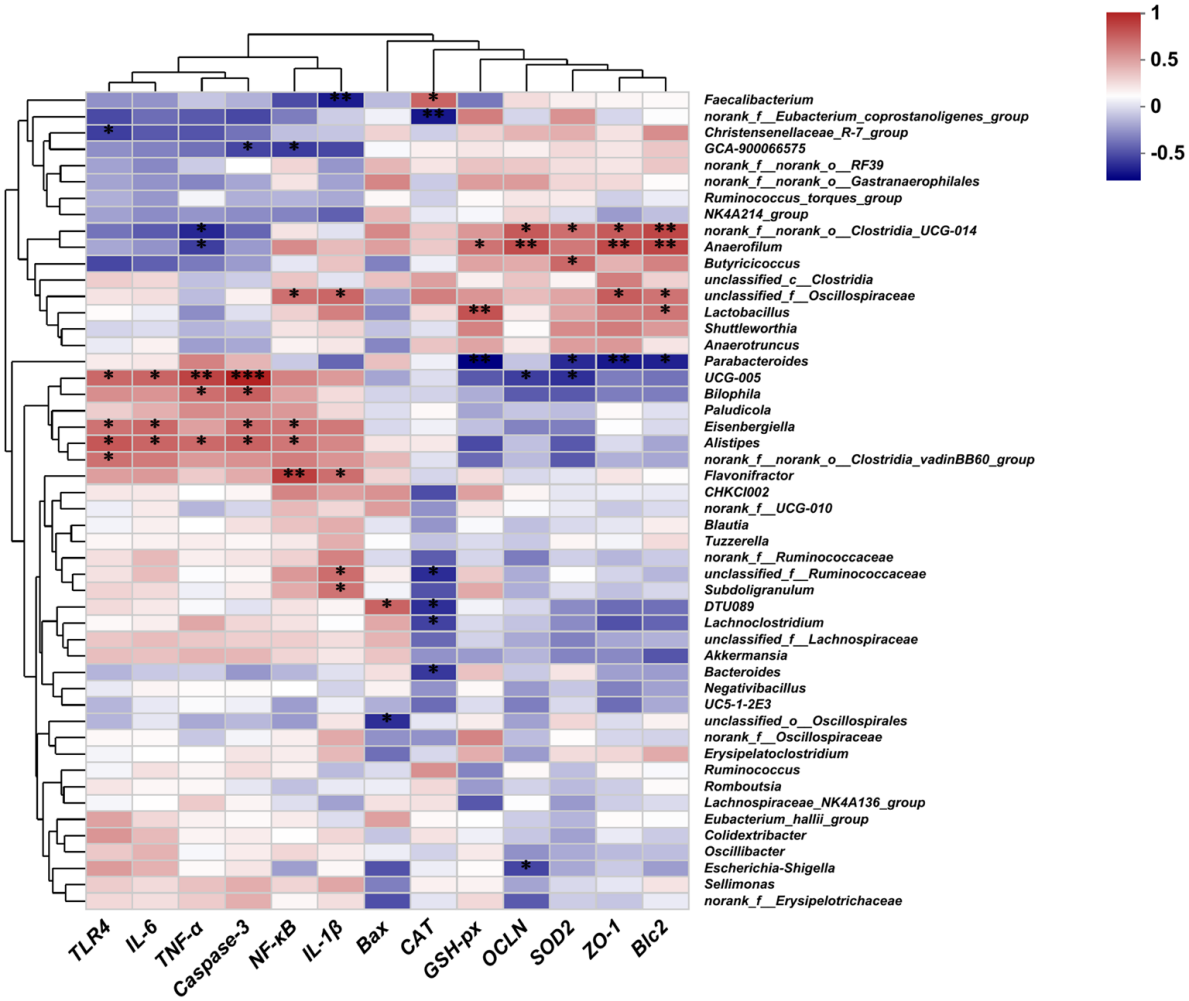
Heatmaps of the Spearman correlation between the gut microbiota and the expression of genes related to gut inflammation, oxidative stress, and the intestinal barrier; *p < 0.05, **p < 0.01, ***p < 0.001.

Spearman correlation analysis was performed to investigate the correlation between the gut microbiota and the expression of genes related to gut inflammation, oxidative stress, and intestinal barrier. As shown in Figure 8, the abundance of *Faecalibacterium*, *Clostridia_UCG-014*, *Anaerofilum*, *Butyricicoccus*, and *Lactobacillus* was positively correlated with the expression of antioxidant genes (*CAT*, *GSH-px*, *SOD2*), intestinal tight junction genes (*ZO-1*, *OCLN*), and anti-apoptotic genes (*Bcl2*), while exhibiting a negative correlation with the expression of inflammatory genes (*IL-1β*, *TNF-α*). The abundance of *UCG-005*, *Bilophila*, *Eisenbergiella*, *Alistipes*, *Clostridia_vadinBB60_group*, unclassified *Ruminococcaceae*, and *DTU089* was positively correlated with the expression of inflammatory cytokines (*TLR4*, *IL-6*, *TNF-α*, *NF-κB*) and apoptotic genes (*Caspase-3*), and negatively associated with the expression of *OCLN* and *SOD2*.

## 4. Discussion

Bacterial LPS challenge induces immune and oxidative stress in animals, which subsequently affects energy utilization and decreases animal growth performance (Liu et al., 2015; Shi et al., 2022). Similarly, we found that LPS challenge reduced the growth performance of chickens, as evidenced by the decreased ADFI and ADG, and the increased feed-to-gain ratio. Dietary 10-HDA supplementation reduced the feed-to-gain ratio and attenuated the growth performance loss induced by LPS in chickens; however, high supplementation doses of 10-HDA were not superior to low supplementation doses. A previous study demonstrated that 10-HDA supplementation improved the growth performance of broiler chickens by increasing the ADG on days 22–42 and 0–42 (Zhang et al., 2022). Here, the addition of 10-HDA did not have a significant effect on the ADFI and ADG in chickens challenged with LPS, which may be attributed to various factors such as the breed or age of the chickens, as well as the ingredients of their diet.

The intestinal mucosal barrier is essential for maintaining homeostasis and preventing intestinal inflammation (Okumura and Takeda, 2018). LPS can induce intestinal inflammation, disrupt the structural integrity of the intestinal wall, and impair intestinal barrier function (Wu et al., 2021). Damage of the intestinal mucosal barrier increases serum DAO activity, which is considered an intestinal permeability marker (Jiang et al., 2019). Here, dietary supplementation with CTC or 10-HDA effectively reduced the LPS-induced elevation of serum DAO levels. Moreover, intestinal tight junction proteins play key roles in the formation of the intestinal selective permeability barrier, contributing to maintaining the integrity of the intestinal structure (Ren et al., 2022). We found that supplementation with CTC or 10-HDA could ameliorate the downregulation of *ZO-1* and *OCLN* expression in the chickens’ jejunum induced by LPS. The unique architecture of the villus crypt of the intestinal tract plays a crucial role in intestinal health (Tong et al., 2023). A higher VCR is associated with stronger digestive and absorptive capacities in the intestines (Saadatmand et al., 2019). Our results showed that the jejunal and ileal VCR decreased after LPS challenge, whereas supplementation with CTC or 10-HDA reversed this decline.

Cytokines are involved in the pathogenesis of inflammatory intestinal diseases (Neurath, 2014). LPS induces intestinal inflammatory responses in chickens by increasing the levels of proinflammatory cytokines (Wang et al., 2014; Shang et al., 2015; Zhang et al., 2021). TLR4 functions as a receptor for LPS, binding to LPS and initiating the TLR4/NF-κB signaling pathway, thereby facilitating the secretion of proinflammatory cytokines (Park and Lee, 2013). NF-κB is a crucial regulatory factor in inflammatory responses and essential in maintaining immune system homeostasis (Christian et al., 2016). Previous studies on murine macrophage cell lines indicated that 10-HDA inhibits the LPS-induced NF-κB pathway and reduces the release of downstream IL-6 (Sugiyama et al., 2012). Chen Y. F. et al. (2018) demonstrated that oral administration of 10-HDA lowered the serum levels of TNF-α, IL-6, and IL-10 in mice. In the current study, LPS stimulation up-regulated the mRNA expression of *TLR4*, *NF-κB*, *TNF-α*, *IL-1β*, and *IL-6* in chicken jejunal mucosa, and elevated the serum concentration of TNF-α, IL-1β, and IL-6, whereas 0.1% 10-HDA supplementation effectively decreased the expression and secretion of these inflammatory cytokines. However, the mRNA expression of *TLR4*, *NF-κB*, *IL-1β*, *IL-6*, and the serum levels of TNF-α and IL-1β induced by LPS were not reversed by 0.5% 10-HDA supplementation. These results suggest that 0.1% 10-HDA supplementation can effectively inhibit intestinal inflammation to attenuate LPS-induced intestinal damage in chickens.

In addition, LPS challenge induces oxidative stress, leading to intestinal injury in chickens (Wu et al., 2013). Antioxidant enzymes could effectively inhibit oxidative damage and reflect the oxidative stress state of animals (Zheng Y. et al., 2020). This study revealed that LPS challenge reduced the activity of T-SOD, CAT, and GSH-px in the serum, and downregulated the expression of the genes encoding these antioxidant enzymes in the jejunal mucosa. Previous studies have shown that 10-HDA increases antioxidant enzyme activity in the liver of diabetic mice and reduces the production of free radicals and lipid peroxidation (Hu X. et al., 2022). Similarly, our results revealed that dietary 0.1% 10-HDA supplementation improved the activities of T-SOD, CAT, and GSH-px in LPS-challenged chickens, indicating that 10-HDA exerts an antioxidant function to maintain the intestinal health of chickens. No significant differences were observed in the activities of CAT and T-SOD between the 0.1% 10-HDA and CTC groups. Moreover, intestinal inflammation and oxidative stress can trigger excessive apoptosis in intestinal mucosal cells and inhibit cell proliferation, thereby destroying intestinal barrier function (Jiang et al., 2019; Chang et al., 2020). We found that the addition of 0.1% 10-HDA to the diet inhibited LPS-induced apoptosis by downregulating the mRNA expression of *Caspase-3* and *Bax* and increasing *Bcl2* expression.

The intestinal microbiota plays key roles in preventing pathogen invasion and regulating intestinal homeostasis, which is essential for maintaining the intestinal health of chickens (Carrasco et al., 2019). LPS stimulation disrupts the ecological balance of the gut microbiota (Lucke et al., 2018; He et al., 2022; Ye et al., 2023). Our PCoA and PLS-DA results demonstrated a significant separation of the gut microbiota between the LPS and control groups. However, the addition of 10-HDA attenuated these differences and improved the compositional structure of the cecal microbiota. 10-HDA treatment increased the abundance of *Faecalibacterium* and *Clostridia_UCG-014*, while reducing the abundance of *Clostridia_vadinBB60_group*, *Eubacterium_nodatum*, and *UC5-1-2E3. Faecalibacterium* is closely related to butyrate production in the chicken intestinal tract and plays a crucial role in anti-inflammation and recovery from intestinal injury (Singh et al., 2014; Breyner et al., 2017; Sun Y. et al., 2022). Our study revealed that the abundance of *Faecalibacterium* was negatively correlated with the expression of *IL-1β* and positively correlated with *CAT* levels. The probiotic genus *Clostridia_UCG-014* is associated with tryptophan metabolism and inflammation. It has been shown to be beneficial in treating disorders of glucose and lipid metabolism (Hu Q. et al., 2022; Niu et al., 2022). *Clostridia_vadinBB60_group* is positively correlated with inflammatory markers and negatively correlated with anti-inflammatory markers (Sun J. et al., 2022). *Eubacterium_nodatum* is an opportunistic pathogen associated with chronic inflammation (Moen et al., 2006). It has been reported that a pro-inflammatory diet is linked to a higher abundance of *Eubacterium_nodatum* (Zheng J. et al., 2020). Furthermore, *Eubacterium_nodatum* is positively correlated with intestinal disorder indices in DSS-induced colitis mice (Ma et al., 2022). Circos analysis revealed that *Alistipes* was the dominant genus in the LPS group, and the addition of 10-HDA reduced its abundance. *Alistipes* is an opportunistic pathogen associated with inflammation and dysbiosis (Kong et al., 2019; Parker et al., 2020). Our study demonstrated a significant positive correlation between the abundance of *Alistipes* and the expression of *TLR4*, *NF-κB*, *TNF-α*, *IL-6*, and *Caspase-3*. Overall, the results indicate that 10-HDA supplementation can alter the cecal microbial composition in LPS-challenged chickens. This alteration may contribute to an enhancement in antioxidant capacity and a reduction in LPS-induced intestinal inflammation and injury.

## 5. Conclusion

In conclusion, 10-HDA alleviated the LPS-induced intestinal damage and growth performance loss in chickens through anti-inflammatory, antioxidant, and gut microbiota-modulating activities. The alterations in the cecal microbiota caused by 10-HDA supplementation might be related to the recovery of intestinal health in chickens subjected to LPS challenge. Moreover, dietary supplementation with 0.1% 10-HDA had comparable or even better protection for LPS-challenged chickens than supplementation with antibiotics or 0.5% 10-HDA. This study provides valuable references for the application of 10-HDA as an alternative to antibiotics in protecting the intestinal health and improving the performance of poultry.

## Data availability statement

The datasets presented in this study can be found in online repositories. The names of the repository/repositories and accession number(s) can be found at: https://www.ncbi.nlm.nih.gov/, PRJNA1000644.

## Ethics statement

The animal study was approved by Animal Ethics committee of Shandong Academy of Agricultural Sciences. The study was conducted in accordance with the local legislation and institutional requirements.

## Author contributions

LH: Conceptualization, Methodology, Data curation, Investigation, Writing – original draft. MZ: Writing – original draft, Investigation, Validation, Writing – original draft. FL: Investigation, Conceptualization, Resources, Writing – review & editing. JS: Investigation, Validation, Writing – original draft. RW: Conceptualization, Resources, Supervision, Writing – review & editing. GL: Conceptualization, Data curation, Investigation, Writing – original draft. XY: Funding acquisition, Writing – review & editing, Conceptualization, Methodology, Project administration.

## Footnotes

^1^https://cloud.majorbio.com

## References

[ref1] Adedokun S. A. Olojede O. C. (2019). Optimizing gastrointestinal integrity in poultry: the role of nutrients and feed additives. Front. Vet. Sci. 5:348. doi: 10.3389/fvets.2018.00348, PMID: 30766877 PMC6366008

[ref2] Bacanli M. Basaran N. (2019). Importance of antibiotic residues in animal food. Food Chem. Toxicol. 125, 462–466. doi: 10.1016/j.fct.2019.01.033, PMID: 30710599

[ref3] Breyner N. M. Michon C. de Sousa C. S. Boas P. B. V. Chain F. Azevedo V. A.. (2017). Microbial anti-inflammatory molecule (mam) from *Faecalibacterium prausnitzii* shows a protective effect on dnbs and DSS-induced colitis model in mice through inhibition of NF-kappa B pathway. Front. Microbiol. 8:114. doi: 10.3389/fmicb.2017.00114, PMID: 28203226 PMC5285381

[ref4] Carrasco J. M. D. Casanova N. A. Miyakawa M. E. F. (2019). Microbiota, gut health and chicken productivity: what is the connection? Microorganisms 7:374. doi: 10.3390/microorganisms7100374, PMID: 31547108 PMC6843312

[ref5] Chang Y. C. Yuan L. Liu J. R. Ishfaq M. Cao C. B. Shi C. X.. (2020). Dihydromyricetin attenuates *Escherichia coli* lipopolysaccharide-induced ileum injury in chickens by inhibiting NLRP3 inflammasome and TLR4/NF-kappa B signalling pathway. Vet. Res. 51:72. doi: 10.1186/s13567-020-00796-8, PMID: 32448367 PMC7247275

[ref6] Chen Y. F. You M. M. Liu Y. C. Shi Y. Z. Wang K. Lu Y. Y.. (2018). Potential protective effect of trans-10-hydroxy-2-decenoic acid on the inflammation induced by lipoteichoic acid. J. Funct. Foods 45, 491–498. doi: 10.1016/j.jff.2018.03.029

[ref7] Chen Y. P. Zhang H. Cheng Y. F. Li Y. Wen C. Zhou Y. M. (2018). Dietary L-threonine supplementation attenuates lipopolysaccharide-induced inflammatory responses and intestinal barrier damage of broiler chickens at an early age. Br. J. Nutr. 119, 1254–1262. doi: 10.1017/s0007114518000740 29770758

[ref8] Christian F. Smith E. L. Carmody R. J. (2016). The regulation of NF-kappa B subunits by phosphorylation. Cells 5:12. doi: 10.3390/cells5010012, PMID: 26999213 PMC4810097

[ref9] Fan P. Han B. Hu H. Wei Q. H. Zhang X. F. Meng L. F.. (2020). Proteome of thymus and spleen reveals that 10-hydroxydec-2-enoic acid could enhance immunity in mice. Expert Opin. Ther. Targets 24, 267–279. doi: 10.1080/14728222.2020.1733529 32077781

[ref10] Gao K. K. Su B. Dai J. Li P. W. Wang R. M. Yang X. H. (2022). Anti-biofilm and anti-hemolysis activities of 10-hydroxy-2-decenoic acid against staphylococcus aureus. Molecules 27:1485. doi: 10.3390/molecules27051485, PMID: 35268586 PMC8912057

[ref11] Gui H. Song I. B. Han H. J. Lee N. Y. Cha J. Y. Son Y. K.. (2018). Antioxidant activity of royal jelly hydrolysates obtained by enzymatic treatment. Korean J. Food Sci. Anim. Resour. 38, 135–142. doi: 10.5851/kosfa.2018.38.1.135, PMID: 29725231 PMC5932976

[ref12] He Z. T. Li Y. J. Xiong T. D. Nie X. Y. Zhang H. H. Zhu C. (2022). Effect of dietary resveratrol supplementation on growth performance, antioxidant capacity, intestinal immunity and gut microbiota in yellow-feathered broilers challenged with lipopolysaccharide. Front. Microbiol. 13:977087. doi: 10.3389/fmicb.2022.977087, PMID: 36090096 PMC9453244

[ref13] Hu X. Y. Liu Z. G. Lu Y. T. Chi X. P. Han K. Wang H. F.. (2022). Glucose metabolism enhancement by 10-hydroxy-2-decenoic acid via the PI3K/AKT signaling pathway in high-fat-diet/streptozotocin induced type 2 diabetic mice. Food Funct. 13, 9931–9946. doi: 10.1039/d1fo03818d, PMID: 36056641

[ref14] Hu Q. Wu C. Y. Yu J. T. Luo J. M. Peng X. C. (2022). Angelica sinensis polysaccharide improves rheumatoid arthritis by modifying the expression of intestinal Cldn5, Slit3 and Rgs18 through gut microbiota. Int. J. Biol. Macromol. 209, 153–161. doi: 10.1016/j.ijbiomac.2022.03.090, PMID: 35318077

[ref15] Huang S. S. Tao R. R. Zhou J. F. Qian L. X. Wu J. (2022). Trans-10-hydroxy-2-decenoic acid alleviates dextran sulfate sodium-induced colitis in mice via regulating the inflammasome-mediated pyroptotic pathway and enhancing colonic barrier function. Mol. Nutr. Food Res. 66:e2100821. doi: 10.1002/mnfr.202100821, PMID: 35373915

[ref16] Jiang J. L. Qi L. N. Lv Z. P. Jin S. Wei X. H. Shi F. X. (2019). Dietary stevioside supplementation alleviates lipopolysaccharide-induced intestinal mucosal damage through anti-inflammatory and antioxidant effects in broiler chickens. Antioxidants 8:575. doi: 10.3390/antiox8120575, PMID: 31766443 PMC6943682

[ref17] Kong C. Gao R. Y. Yan X. B. Huang L. S. Qin H. L. (2019). Probiotics improve gut microbiota dysbiosis in obese mice fed a high fat or high-sucrose diet. Nutrition 60, 175–184. doi: 10.1016/j.nut.2018.10.002 30611080

[ref18] Leshchinsky T. V. Klasing K. C. (2001). Divergence of the inflammatory response in two types of chickens. Dev. Comp. Immunol. 25, 629–638. doi: 10.1016/S0145-305X(01)00023-4, PMID: 11472784

[ref19] Li Y. Wang J. Q. Wang F. Wang L. Wang L. L. Xu Z. Q.. (2022). Production of 10-hydroxy-2-decenoic acid from decanoic acid via whole-cell catalysis in engineered *Escherichia coli*. ChemSusChem 15:e202102152. doi: 10.1002/cssc.202102152, PMID: 34796684

[ref20] Liu L. Qin D. K. Wang X. F. Feng Y. Yang X. J. Yao J. H. (2015). Effect of immune stress on growth performance and energy metabolism in broiler chickens. Food Agric. Immunol. 26, 194–203. doi: 10.1080/09540105.2014.882884

[ref21] Liu C. S. Zhao D. F. Ma W. J. Guo Y. D. Wang A. J. Wang Q. L.. (2016). Denitrifying sulfide removal process on high-salinity wastewaters in the presence of *Halomonas sp*. Appl. Microbiol. Biotechnol. 100, 1421–1426. doi: 10.1007/s00253-015-7039-6, PMID: 26454867

[ref22] Lucke A. Bohm J. Zebeli Q. Metzler-Zebeli B. U. (2018). Dietary deoxynivalenol contamination and oral lipopolysaccharide challenge alters the cecal microbiota of broiler chickens. Front. Microbiol. 9:804. doi: 10.3389/fmicb.2018.00804, PMID: 29922239 PMC5996912

[ref23] Ma L. Zhao X. Liu T. Wang Y. Wang J. Kong L.. (2022). Xuanfei baidu decoction attenuates intestinal disorders by modulating NF-κB pathway, regulating T cell immunity and improving intestinal flora. Phytomedicine 101:154100. doi: 10.1016/j.phymed.2022.154100, PMID: 35489324

[ref24] Mehdi Y. Letourneau-Montminy M. P. Gaucher M. L. Chorfi Y. Suresh G. Rouissi T.. (2018). Use of antibiotics in broiler production: global impacts and alternatives. Anim. Nutr. 4, 170–178. doi: 10.1016/j.aninu.2018.03.002, PMID: 30140756 PMC6103476

[ref25] Melliou E. Chinou I. (2005). Chemistry and bioactivity of royal jelly from Greece. J. Agric. Food Chem. 53, 8987–8992. doi: 10.1021/jf051550p 16277392

[ref26] Moen K. Brun J. G. Valen M. Skartveit L. Eribe E. K. R. Olsen I.. (2006). Synovial inflammation in active rheumatoid arthritis and psoriatic arthritis facilitates trapping of a variety of oral bacterial DNAs. Clin. Exp. Rheumatol. 24, 656–663. PMID: 17207381

[ref27] Neurath M. F. (2014). Cytokines in inflammatory bowel disease. Nat. Rev. Immunol. 14, 329–342. doi: 10.1038/nri3661 24751956

[ref28] Niu K. X. Bai P. P. Yang B. B. Feng X. C. Qiu F. (2022). Asiatic acid alleviates metabolism disorders in Ob/Ob mice: mechanistic insights. Food Funct. 13, 6934–6946. doi: 10.1039/d2fo01069k, PMID: 35696250

[ref29] Okumura R. Takeda K. (2018). Maintenance of intestinal homeostasis by mucosal barriers. Inflamm. Regen. 38:5. doi: 10.1186/s41232-018-0063-z, PMID: 29619131 PMC5879757

[ref30] Park B. S. Lee J. O. (2013). Recognition of lipopolysaccharide pattern by TLR4 complexes. Exp. Mol. Med. 45:e166:e66. doi: 10.1038/emm.2013.97, PMID: 24310172 PMC3880462

[ref31] Parker B. J. Wearsch P. A. Veloo A. C. M. Rodriguez-Palacios A. (2020). The genus *Alistipes*: gut bacteria with emerging implications to inflammation, cancer, and mental health. Front. Immunol. 11:906. doi: 10.3389/fimmu.2020.00906, PMID: 32582143 PMC7296073

[ref32] Ren Z. S. Fang H. T. Zhang J. Wang R. Xiao W. Y. Zheng K. X.. (2022). Dietary aronia melanocarpa pomace supplementation enhances the expression of ZO-1 and occludin and promotes intestinal development in pigs. Front. Vet. Sci. 9:904667. doi: 10.3389/fvets.2022.904667, PMID: 35711808 PMC9196908

[ref33] Robles-Jimenez L. E. Aranda-Aguirre E. Castelan-Ortega O. A. Shettino-Bermudez B. S. Ortiz-Salinas R. Miranda M.. (2022). Worldwide traceability of antibiotic residues from livestock in wastewater and soil: a systematic review. Animals 12:60. doi: 10.3390/ani12010060, PMID: 35011166 PMC8749557

[ref34] Saadatmand N. Toghyani M. Gheisari A. (2019). Effects of dietary fiber and threonine on performance, intestinal morphology and immune responses in broiler chickens. Anim. Nutr. 5, 248–255. doi: 10.1016/j.aninu.2019.06.001 31528726 PMC6739262

[ref35] Shang Y. Regassa A. Kim J. H. Kim W. K. (2015). The effect of dietary fructooligosaccharide supplementation on growth performance, intestinal morphology, and immune responses in broiler chickens challenged with salmonella enteritidis lipopolysaccharides. Poult. Sci. 94, 2887–2897. doi: 10.3382/ps/pev275 26467012

[ref36] Shi L. L. Jin X. Xu Y. Q. Xing Y. Y. Yan S. M. Guo Y. F.. (2022). Effects of total flavonoids of artemisia ordosica on growth performance, oxidative stress, and antioxidant status of lipopolysaccharide-challenged broilers. Antioxidants 11:1985. doi: 10.3390/antiox11101985, PMID: 36290707 PMC9598371

[ref37] Singh N. Gurav A. Sivaprakasam S. Brady E. Padia R. Shi H. D.. (2014). Activation of gpr109a, receptor for niacin and the commensal metabolite butyrate, suppresses colonic inflammation and carcinogenesis. Immunity 40, 128–139. doi: 10.1016/j.immuni.2013.12.007, PMID: 24412617 PMC4305274

[ref38] Sugiyama T. Takahashi K. Tokoro S. Gotou T. Neri P. Mori H. (2012). Inhibitory effect of 10-hydroxy-trans-2-decenoic acid on LPS-induced IL-6 production via reducing I kappa B-zeta expression. Innate Immun. 18, 429–437. doi: 10.1177/1753425911416022 21948282

[ref39] Sun J. Liu J. Ren G. Chen X. T. Cai H. H. Hong J. H.. (2022). Impact of purple sweet potato (*Ipomoea batatas l.*) polysaccharides on the fecal metabolome in a murine colitis model. RSC Adv. 12, 11376–11390. doi: 10.1039/d2ra00310d 35425052 PMC9004255

[ref40] Sun Y. G. Zhang S. S. Nie Q. X. He H. J. Tan H. Z. Geng F.. (2022). Gut firmicutes: relationship with dietary fiber and role in host homeostasis. Crit. Rev. Food Sci. Nutr. 12, 1–16. doi: 10.1080/10408398.2022.2098249, PMID: 35822206

[ref41] Tao W. J. Wang G. Pei X. Sun W. J. Wang M. Q. (2022). Chitosan oligosaccharide attenuates lipopolysaccharide-induced intestinal barrier dysfunction through suppressing the inflammatory response and oxidative stress in mice. Antioxidants 11:1384. doi: 10.3390/antiox11071384, PMID: 35883875 PMC9312058

[ref42] Tong Y. C. Yu C. Y. Chen S. Zhang X. L. Yang Z. B. Wang T. (2023). Trans-anethole exerts protective effects on lipopolysaccharide-induced acute jejunal inflammation of broilers via repressing NF-κB signaling pathway. Poult. Sci. 102:102397. doi: 10.1016/j.psj.2022.102397, PMID: 36565631 PMC9801195

[ref43] Vancamelbeke M. Vermeire S. (2017). The intestinal barrier: a fundamental role in health and disease. Expert Rev.Gastroenterol.Hepatol. 11, 821–834. doi: 10.1080/17474124.2017.1343143, PMID: 28650209 PMC6104804

[ref44] Wang X. F. Shen J. Li S. Z. Zhi L. H. Yang X. J. Yao J. H. (2014). Sulfated astragalus polysaccharide regulates the inflammatory reaction in LPS-infected broiler chicks. Int. J. Biol. Macromol. 69, 146–150. doi: 10.1016/j.ijbiomac.2014.05.004, PMID: 24820152

[ref45] Wu Y. P. Li Q. Liu J. S. Liu Y. L. Xu Y. L. Zhang R. Q.. (2021). Integrating serum metabolome and gut microbiome to evaluate the benefits of lauric acid on lipopolysaccharide- challenged broilers. Front. Immunol. 12:759323. doi: 10.3389/fimmu.2021.759323, PMID: 34721434 PMC8554146

[ref46] Wu Q. J. Zhou Y. M. Wu Y. N. Zhang L. L. Wang T. (2013). The effects of natural and modified clinoptilolite on intestinal barrier function and immune response to LPS in broiler chickens. Vet. Immunol. Immunopathol. 153, 70–76. doi: 10.1016/j.vetimm.2013.02.006, PMID: 23453767

[ref47] Yang Y. C. Chou W. M. Widowati D. A. Lin I. P. Peng C. C. (2018). 10-Hydroxy-2-decenoic acid of royal jelly exhibits bactericide and anti-inflammatory activity in human colon cancer cells. BMC Complement. Altern. Med. 18:202. doi: 10.1186/s12906-018-2267-9, PMID: 29970062 PMC6029378

[ref48] Yang X. J. Li W. L. Feng Y. Yao J. H. (2011). Effects of immune stress on growth performance, immunity, and cecal microflora in chickens. Poult. Sci. 90, 2740–2746. doi: 10.3382/ps.2011-01591, PMID: 22080012

[ref49] Ye J. X. Yang H. A. Hu W. D. Tang K. Y. Liu A. F. Bi S. C. (2023). Changed cecal microbiota involved in growth depression of broiler chickens induced by immune stress. Poult. Sci. 102:102598. doi: 10.1016/j.psj.2023.102598, PMID: 36913756 PMC10023976

[ref50] You M. M. Miao Z. N. Tian J. Hu F. L. (2020). Trans-10-hydroxy-2-decenoic acid protects against LPS-induced neuroinflammation through FOXO1-mediated activation of autophagy. Eur. J. Nutr. 59, 2875–2892. doi: 10.1007/s00394-019-02128-9, PMID: 31820078

[ref51] Zhang Y. X. Geng S. X. Di Y. T. Sun Y. B. Liu Y. Li J. T.. (2022). 10-Hydroxy-trans-2-decenoic acid, a new potential feed additive for broiler chickens to improve growth performance. Animals 12:1846. doi: 10.3390/ani12141846, PMID: 35883394 PMC9311973

[ref52] Zhang J. F. Yang Y. X. Han H. L. Zhang L. L. Wang T. (2021). Bisdemethoxycurcumin attenuates lipopolysaccharide-induced intestinal damage through improving barrier integrity, suppressing inflammation, and modulating gut microbiota in broilers. J. Anim. Sci. 99:skab296. doi: 10.1093/jas/skab296, PMID: 34664650 PMC8598923

[ref53] Zhang S. F. Zhou Q. N. Li Y. C. Zhang Y. L. Wu Y. M. (2020). Mitoq modulates lipopolysaccharide-induced intestinal barrier dysfunction via regulating Nrf2 signaling. Mediat. Inflamm. 2020:3276148. doi: 10.1155/2020/3276148, PMID: 32351320 PMC7171662

[ref54] Zheng J. L. Hoffman K. L. Chen J. S. Shivappa N. Sood A. Browman G. J.. (2020). Dietary inflammatory potential in relation to the gut microbiome: results from a cross-sectional study. Br. J. Nutr. 124, 931–942. doi: 10.1017/s0007114520001853, PMID: 32475373 PMC7554089

[ref55] Zheng Y. W. Zhang J. Y. Zhou H. B. Guo Y. P. Ma Q. G. Ji C.. (2020). Effects of dietary pyrroloquinoline quinone disodium supplementation on inflammatory responses, oxidative stress, and intestinal morphology in broiler chickens challenged with lipopolysaccharide. Poult. Sci. 99, 5389–5398. doi: 10.1016/j.psj.2020.08.007, PMID: 33142455 PMC7647834

