# Supplementary material for: 10-hydroxy-2-decenoic acid alleviates lipopolysaccharide-induced intestinal mucosal injury through anti-inflammatory, antioxidant, and gut microbiota modulation activities in chickens
Source: Front Microbiol. 2023 Oct 17;14:1285299. doi: 10.3389/fmicb.2023.1285299 (PMC10616258; doi:10.3389/fmicb.2023.1285299)
Supplement: Supplementary file 1 [file Data_Sheet_1.docx]

Supplementary Material

# Supplementary Table 1. The composition and nutrient levels of basal diet.

| Ingredients | Content (%) | Nutrient levels | Content |
| --- | --- | --- | --- |
| Corn | 62.85 | Metabolizable energy, MJ/kg | 12.08 |
| Soybean meal | 31.50 | Crude protein, % | 19.00 |
| Soybean oil | 1.30 | Calcium, % | 0.95 |
| Limestone | 1.50 | Available phosphorus, % | 0.41 |
| CaHPO_4_ | 1.50 | Lysine, % | 0.92 |
| NaCl | 0.35 | Methionine, % | 0.39 |
| Premix | 1.00 |  |  |
| Total | 100.00 |  |  |

The premix provided the following per kg of the diet: Cu 5 mg, Fe 75.0 mg, Mn 56.0 mg, I 0.35 mg, Se 0.14 mg, Zn 38 mg, VA 1420 IU, VD 2190 IU, VE 9.5 IU, VK 0.38 mg, riboflavin 3.4 mg, pantothenic acid 9.4 mg, nicotinic acid 26.0 mg, VB_12_ 0.009 mg, choline 1225 mg, biotin 0.14 mg, folic acid 0.52 mg, thioflavin 1.0 mg, pyridoxic acid 2.8 mg.

**Supplementary** **Table 2.** List of used primers for RT-qPCR.

| Gene | Primer Sequence (5' to 3') | Length (bp) | GenBank accession |
| --- | --- | --- | --- |
| *TLR4* | F: AGGCACCTGAGCTTTTCCTC  R: TACCAACGTGAGGTTGAGCC | 96 | NM_001030693.1 |
| *NK-κB* | F: GTGTGAAGAAACGGGAACTG  R: GGCACGGTTGTCATAGATGG | 203 | NM_205129.1 |
| TNF-α | F: TGGCAGCTGTGGTGCAAATA  R: TGCAGCCTTTGCAGAGATGA | 125 | NM_204267.2 |
| *IL-1β* | F: GTACCGAGTACAACCCCTGC  R: AGCAACGGGACGGTAATGAA | 112 | NM_204524.1 |
| *IL-6* | F: AAATCCCTCCTCGCCAATCT  R: CCCTCACGGTCTTCTCCATAAA | 106 | NM_204628.1 |
| *CAT* | F: GTTGGCGGTAGGAGTCTGGTCT  R: GTGGTCAAGGCATCTGGCTTCTG | 182 | NM_001031215.2 |
| *GSH-px* | F: TCACCATGTTCGAGAAGTGC  R: ATGTACTGCGGGTTGGTCAT | 124 | NM_001277853.2 |
| *SOD2* | F: CAGATAGCAGCCTGTGCAAATCA  R: GCATGTTCCCATACATCGATTCC | 86 | NM_204211.1 |
| *Bax* | F: TCCTCATCGCCATGCTCAT  R: CCTTGGTCTGGAAGCAGAAGA | 69 | XM_046922136.1 |
| *Caspase-3* | F: CGGACTGTCATCTCGTTCA  R: TGGCTTAGCAACACACAAAC | 186 | XM_046915477.1 |
| *Bcl2* | F: GATCGTCGCCTTCTTCGAGT  R: GGCCTCATACTGTTGCCGTA | 186 | NM_205339.3 |
| *ZO-1* | F: CTTCAGGTGTTTCTCTTCCTCCTC  R: CTGTGGTTTCATGGCTGGATC | 131 | XM_015278975.2 |
| *OCLN* | F: ACGGCAGCACCTACCTCAA  R: GGGCGAAGAAGCAGATGAG | 123 | NM_205128.1 |
| *β-actin* | F: TGTTACCAACACCCACACCC  R: TCCTGAGTCAAGCGCCAAAA | 110 | NM_205518.1 |

TLR4, toll-like receptor 4; NF-κB, nuclear factor kappa B; TNF-α, tumor necrosis factor-alpha; IL-1β, interleukin 1 beta; IL-6, interleukin 6; CAT, catalase; GSH-px, glutathione peroxidase; SOD2, superoxide dismutase-2; Bax, Bcl2-associated X; Bcl2, B-cell lymphoma-2; ZO-1, zonula occludens-1; OCLN, occludin.
